# Structural Phase Transition and the Effect of Iodine on Phase Stability in Rb_3_Bi_2_Br_9_ Perovskite-Related Halides with 2D Dimensionality

**DOI:** 10.1021/acs.inorgchem.5c03701

**Published:** 2025-10-21

**Authors:** Yousra Chakroun, Wajdi Cherif, Carlos A. López, Brenda Martinelli, Francielen S. M. Rodrigues, Federico Serrano-Sánchez, Javier Gainza, Romualdo S. Silva, Mateus M. Ferrer, José Luis Martinez, Maria Teresa Fernández-Díaz, João Elias F. S. Rodrigues, José Antonio Alonso

**Affiliations:** † 69570 Instituto de Ciencia de Materiales de Madrid, CSIC, Cantoblanco, Madrid 28049, Spain; ‡ Laboratory Inorganic Chemistry, Faculty of Sciences of Sfax, 198828 University of Sfax, Sfax 3000, Tunisia; § National Engineering School of Sfax (ENIS), Laboratory of Electromechanical Systems (LASEM), B.P.W., Sfax 3038, Tunisia; ∥ Instituto de Investigaciones en Tecnología Química (UNSL-CONICET) and Facultad de Química, Bioquímica y Farmacia, Almirante Brown, San Luis 1455 (5700), Argentina; ⊥ CCAF, PPGCEM/CDTec, 37902 Federal University of Pelotas, Pelotas 96010-610, Rio Grande do Sul, Brazil; # 55553 European Synchrotron Radiation Facility (ESRF), 71 Avenue des Martyrs, Grenoble 38000, France; ¶ 56053 Institut Laue Langevin, BP 156X, Grenoble F-38042, France

## Abstract

Rubidium-based halide perovskites Rb_3_Bi_2_Br_9–*x*_I_*x*_ (*x* = 0, 3) represent a lead-free, low-dimensional alternative within the A_3_B_2_X_9_ family, offering promising optoelectronic properties. This work reports the successful synthesis of Rb_3_Bi_2_Br_9_ and Rb_3_Bi_2_Br_6_I_3_ via mechanochemical ball milling, yielding highly crystalline products. Structural characterization of Rb_3_Bi_2_Br_9_ halide, using synchrotron X-ray and neutron powder diffraction across a broad temperature range (295–656 K), revealed a reversible phase transition from a low-symmetry monoclinic (space group: *P*2_1_/*c*) to a high-symmetry trigonal (space group: *P*3̅̅*m*1) phase at ∼450 K (both with a 2D dimensionality, concerning the connection of [BiBr_6_] octahedra). Thermal expansion coefficients, derived from unit-cell evolution, showed discontinuity across the structural phase transition. Symmetry-adapted distortion mode analysis identified octahedral tilting and rigid [BiBr_6_] framework rotations as the primary contributors to the monoclinic distortion, with minor and moderate contributions from stretching, bending, and Rb atoms translations. Optical characterization at room conditions (monoclinic phase) unveiled bandgaps of ∼2.70 and ∼2.21 eV for Rb_3_Bi_2_Br_9_ and Rb_3_Bi_2_Br_6_I_3_, respectively. Density functional theory (DFT) calculations corroborated the direct bandgap nature and electronic structure, highlighting the dominant Br and Bi orbital contributions. These results demonstrate the structural richness and optical tunability of Rb_3_Bi_2_Br_9–*x*_I_*x*_, establishing them as robust candidates for stable, lead-free optoelectronic applications.

## Graphical abstract

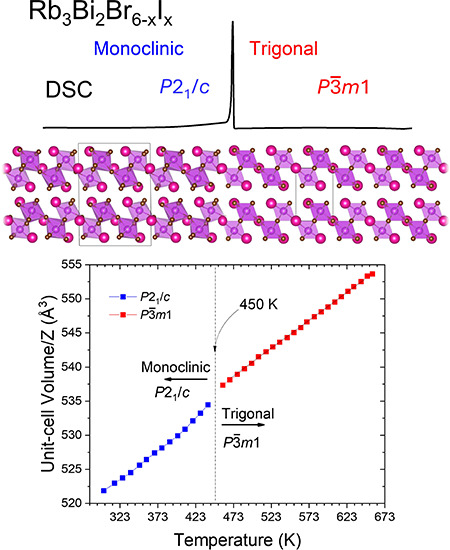

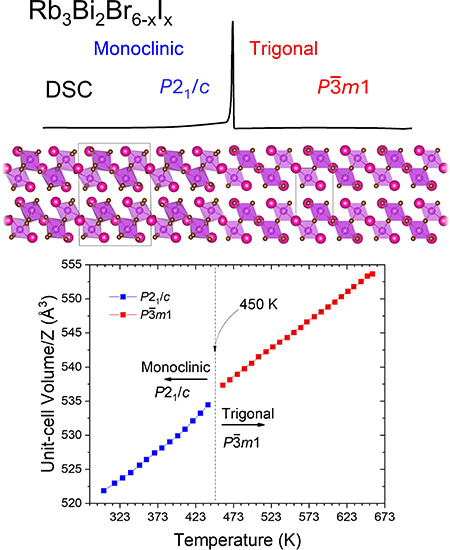

## 1 Introduction

Hybrid halide perovskites have emerged as groundbreaking materials in the field of photovoltaics,^1–9^ demonstrating remarkable potential for next-generation solar cells. These complex halides, typically represented by the formula ABX_3_ [where A is an organic cation-like methylammonium (CH_3_NH_3_^+^), B is a metal cation like lead (Pb^2+^), and X is a halide anion, such as iodide (I^–^), bromide (Br^–^), or chloride (Cl^–^)], combine the advantages of both organic and inorganic semiconductors, such as high charge carrier mobility and long diffusion length.^10–15^ As main advantages, these perovskite-type materials exhibit high power conversion efficiency (PCE), exceeding ∼25%, making them competitive with traditional silicon-based solar cells.^16–19^ This rapid progress, achieved within a decade, highlights the unique optoelectronic properties of perovskites, including high absorption coefficients and tunable bandgaps. The processes employed to manufacture perovskite solar cells are less energy-intensive and cheaper compared to the high-temperature, high-vacuum processes required for silicon photovoltaics. Methods like spin-coating, inkjet printing, vapor deposition enable scalable, and versatile production.^20–27^ Unlike rigid silicon wafers, perovskite solar cells can be fabricated on flexible substrates,^28–31^ making them suitable for a variety of applications, including portable and wearable electronics, building-integrated photovoltaics, and even space applications.

Despite their advantages, hybrid halide perovskites face several challenges that must be addressed for commercial viability: the chemical stability is precarious, and hence perovskite solar cells are sensitive to moisture, oxygen, and UV light,^32–35^ which can degrade the material and reduce cell performance over time. Encapsulation techniques and the development of more stable perovskite compositions are crucial for improving longevity.

An interesting approach is the use of all-inorganic perovskites, like CsPbI_3_ (cesium lead iodide).^36–42^ It has a high absorption coefficient in the visible spectrum, making it an efficient light-harvester for solar cells; this inorganic perovskite exhibits a direct bandgap of 1.73 eV, which is ideal for photovoltaic applications as it enables efficient photon absorption and charge carrier generation. Moreover, CsPbI_3_ also exhibits long carrier diffusion lengths,^43,44^ which are beneficial for efficient charge extraction and minimizing recombination losses. It offers better thermal stability compared to its organic–inorganic counterparts like methylammonium lead iodide (CH_3_NH_3_PbI_3_).^45,46^ This makes it more suitable for applications in environments with varying temperatures. CsPbI_3_ can adopt several crystal structures depending on the temperature. The most sought-after phase for photovoltaic applications is the black cubic phase (α-CsPbI_3_), which possesses an ideal perovskite structure conducive to excellent optoelectronic properties.^36^ However, this phase is stable only at high temperatures and tends to transition to a nonperovskite yellow phase (δ-CsPbI_3_) at room temperature,^47,48^ which has inferior electronic properties. Unfortunately, the use of lead in most high-efficiency perovskites (either hybrid or purely inorganic) raises environmental and health concerns. Researchers are actively exploring lead-free alternatives, such as tin-based perovskites, although these currently suffer from lower efficiencies and stability issues.

Recently, alternative strategies have been developed to design novel materials with appealing properties, which include different topologies for the octahedral arrangements, from 3D (e.g., CsSnBr_3_ ^49^ and Cs_2_AgSbCl_6_ ^50^), where the [BX_6_] octahedra share corners in a three-dimensional framework, to 2D (e.g., CsSn_2_Br_5_ ^51^) to 0D (i.e., without connection between octahedra), as the case of the Cs_4_PbX_6_ halide.^52^ These families contain divalent cations such as Pb^2+^ or Sn^2+^; however, trivalent bismuth, Bi^3+^, can also leads to low-dimensional structures as is the case with Cs_3_Bi_2_Br_9_ phase.^53^ This compound exhibits a trigonal structure and good stability in ambient conditions. The crystal structure of these materials is complex, as many undergo a cascade of phase transitions, so far unexplored. Understanding the interplay between inorganic components in perovskites is crucial for optimizing their properties and advancing their applications in energy devices.

Regarding the family of the mentioned 2D perovskite-related materials A_3_B_2_X_9_ ^54–57^ they cover a wide compositional range, since A can be Cs or Rb; B can be Sb or Bi; and X can be Cl, Br, or I. The trigonal space-group (*P* 3 *m*1) and atomic positions in the crystal structure have been definitively established only for Cs_3_Bi_2_Br_9_ microcrystals.^58^ For other compositions, such as Rb_3_Sb_2_Br_9–*x*_I_*x*_, single-crystal data reveal that all compounds crystallize in a 2D-layered monoclinic crystal structure.^59^ Here, we address the full crystallographic characterization at room temperature of Rb_3_Bi_2_Br_9_ and Rb_3_Bi_2_Br_6_I_3_ from high-resolution synchrotron X-ray (SXRD) and neutron powder diffraction (NPD). We have successfully synthesized these two members of the A_3_B_2_X_9_ perovskite family using a solvent-free mechanochemical method with green credentials. Working under a nitrogen atmosphere during the solid-state reaction ensures the desired phase formation and stability. For Rb_3_Bi_2_Br_9_, temperature-dependent SXRD data revealed a reversible phase transition from a low-symmetry monoclinic (*P*2_1_/*c*) to a high-symmetry trigonal (*P* 3 *m*1) phase at ∼450 K.

## 2 Materials and Methods

### 2.1 Synthesis

Rb_3_Bi_2_Br_9_ and Rb_3_Bi_2_Br_6_I_3_ were synthesized in polycrystalline form by mechanochemical synthesis (ball milling) from stoichiometric amounts of RbBr, RbI, and BiBr_3_. The total mass of reactants was 1.5 g, which were weighted and mixed with 20 zirconia balls (5 mm in diameter) in a N_2_-filled glovebox. The reaction took place in a Retsch PM100 mill for 4 h at 450 rpm, in a sealed zirconia-lined jar with N_2_ atmosphere.

### 2.2 Structural Characterization

Laboratory XRD (X-ray diffraction) patterns were collected in a Bruker D8 diffractometer with Cu Kα (λ = 1.5418 Å) radiation. Room temperature crystal structure determination was performed using synchrotron X-ray diffraction (SXRD) and neutron powder diffraction (NPD) in Rb_3_Bi_2_Br_9_ and Rb_3_Bi_2_Br_6_I_3_. For Rb_3_Bi_2_Br_9_, the thermal evolution of the crystallographic structure was studied by SXRD, in the range 295–656 K (temperature steps of 10 K and stabilization time of 5 min) at the MSPD beamline, equipped with a *Mythen2* detector and selecting an incident beam with λ = 0.44367 Å^60^ in the ALBA Synchrotron Light Source (Cerdanyola del Vallès, Spain). Additional SXRD patterns were also collected at low temperature (10 and 50 K) at the ID22 diffractometer of the ESRF (Grenoble, France) with λ = 0.35429 Å (35 keV) to cross-check the results obtained in ALBA (but the results are not showed here). A Dectris Eiger2 (2M-W CdTe) pixel detector was placed in Position Sensitive Mode to access a large 2θ angular range of 1–40°. In both MSPD and ID22, the sample was contained in a 0.5 mm-diameter borosilicate capillary that was rotating during the data acquisition (300 rpm). NPD patterns were collected at the D2B instrument, located in the Institut Laue-Langevin reactor (Grenoble, France) with a wavelength of 1.594 Å. The powder sample was contained in 6 mm in diameter vanadium holder. SXRD and NPD patterns were analyzed with the Rietveld method using the *FullProf* program.^61,62^

### 2.3 Thermal Analysis

Thermal stabilities were studied from Thermogravimetric Analysis (TGA) and Differential Scanning Calorimetry (DSC). TGA measurements were carried out in air atmosphere from room temperature up to 600 °C. DSC measurements were carried out in the range 130 up to 520 K in a Mettler TA3000 system equipped with a DSC-30 unit. The heating and cooling rates were set to 10 K·min^–1^, using about 70 mg of sample in each run.

### 2.4 Microstructure

Field-Effect Scanning Electron Microscopy (FE-SEM) images were obtained in a FEI-Nova microscope, with an acceleration potential of 5 kV, coupled to an energy-dispersive X-ray spectrometer (EDX), working with an acceleration voltage of 18 kV and 60 s of acquisition time.

### 2.5 Optical Absorption

The optical diffuse reflectance spectrum was measured at room temperature using a UV–vis spectrophotometer Varian-Cary 5000.

### 2.6 Density Functional Theory

Density functional theory (DF*T*) calculations were performed with the *CRYSTAL*23 ^63^ simulation package using the HSE06 hybrid functional.^64^ The triple-ζ-valence with polarization (POB-TZVP) basis sets developed by Laun and co-workers were employed for rubidium (Rb), bismuth (Bi), and bromine (Br) atomic centers.^65^ The precision of the infinite Coulomb and *HF* exchange series was controlled by five α_*k*_ parameters (*k* = 1–5), where α_1_ governs overlap, α_2_ Coulomb integral penetration, α_3_ *HF* exchange integral overlap, and α_4_/α_5_ pseudo-overlaps (*HF*-exchange series). These parameters were set to 8, 8, 8, 8, and 16, respectively. A Pack-Monkhorst grid with shrinking factors 8 and 8 was used for *k*-point sampling, along with the Gilat net for Brillouin zone integration.

## 3 Results

### 3.1 Initial Characterization

The samples were obtained as yellow (Rb_3_Bi_2_Br_9_, hereafter: Br_9_) or orange (Rb_3_Bi_2_Br_3_I_3_, hereafter: Br_6_I_3_) microcrystalline powders; the laboratory XRD patterns at room temperature unveiled a monoclinic symmetry, indexable in the space-group *P*2_1_/*c*. It belongs to the (NH_4_)­Pb_2_Br_5_-structural type.^66^ Figure 1a and Figure 1b exhibit two preliminary Le Bail fits of the laboratory XRD patterns; a large, preferred orientation was observed for the Br_6_I_3_ sample. In Figure 2, the TGA and DSC curves for both samples are illustrated. Both samples are stable up to 590 K. A narrow endothermic event is observed during the heating at 458 and 478 K for Rb_3_Bi_2_Br_9_ and Rb_3_Bi_2_Br_6_I_3_, respectively, which can be assigned to a structural transition to a high symmetry phase (as discussed later).

**Figure 1:**
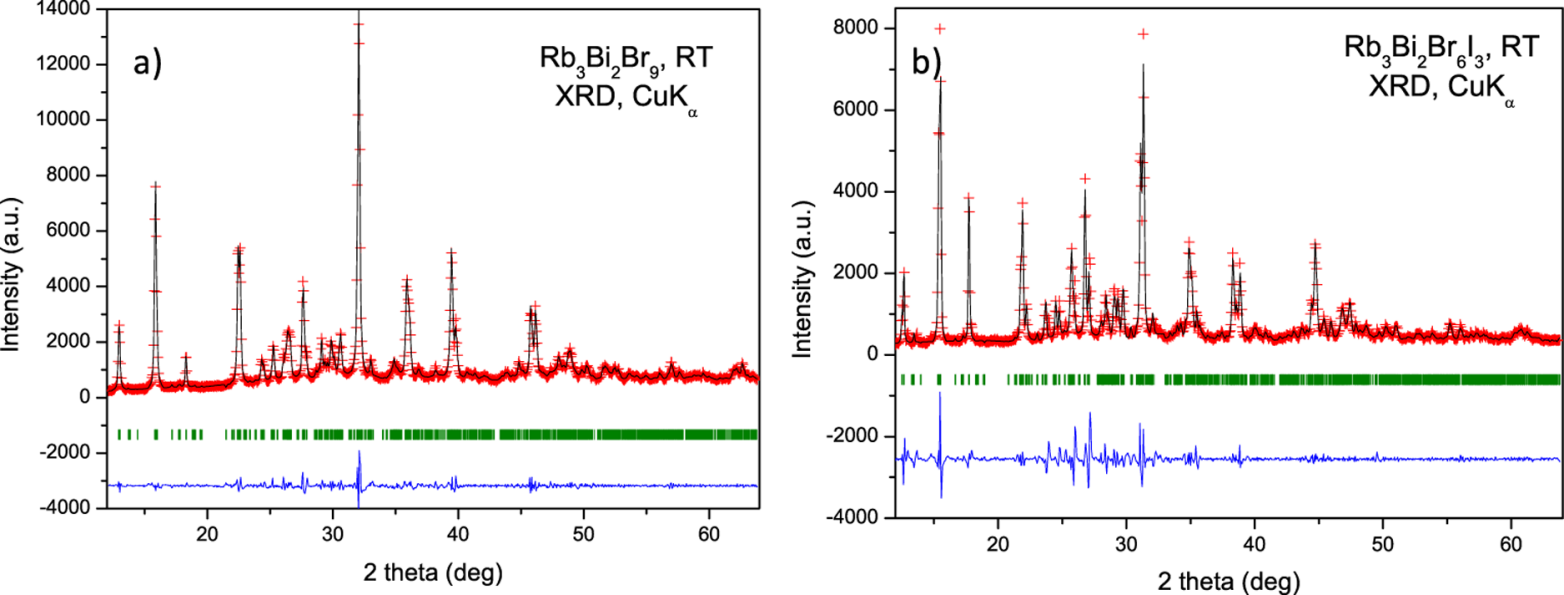
Le Bail refinements of the laboratory XRD patterns of (a) Rb_3_Bi_2_Br_9_ and (b) Rb_3_Bi_2_Br_6_I_3_, collected with Cu Kα radiation at room temperature. Le Bail plot: observed (red crosses) and calculated (black line) X-ray diffraction pattern. Blue lines represent the fit residuals, and the green bars are the expected Bragg reflections.

**Figure 2:**
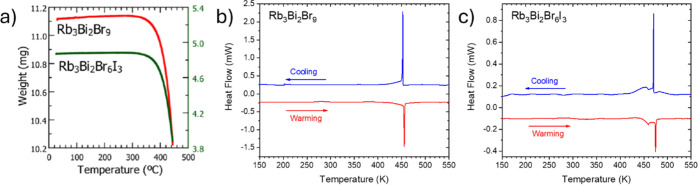
TGA (a) and DSC curves of (b) Rb_3_Bi_2_Br_9_ and (c) Rb_3_Bi_2_Br_6_I_3_, collected in air atmosphere.

FE-SEM images are illustrated for Rb_3_Bi_2_Br_9_, as plotted in Figure 3, giving insight into the microstructure of this product, synthesized using ball milling. An overall view with low magnification (12,500×) shows irregular-shaped clusters of particles of different sizes (see Figure 3a). However, in large magnification views, Figure 3b (50,000×) and Figure 3c (120,000×) unveil that they are indeed formed by tiny crystalline nanocrystals of uneven form, with typical size of 140–170 nm, which are grown during the ball milling process. For the iodine-containing material Rb_3_Bi_2_Br_6_I_9_ (Figure 3d–f) the images show much better crystallized particles of a bigger size, between 1 and 2 μm in average, also forming large agglomerates of particles (Figure 3d). This suggest that the presence of I facilitates the growth of microcrystals. EDX analysis coupled with the FE-SEM images yields an atomic composition close to 3:2:8 for the Rb/Bi/Br ratio, significantly defective in Br, which is probably inherent to the technique. Similar results are obtained for the Rb_3_Bi_2_Br_6_I_3_ composition, with atomic compositions derived from EDX spectra in reasonable agreement with those expected. Typical EDX spectra for Br_9_ and Br_6_I_3_ are included in Figure S1 in the Supporting Information.

**Figure 3:**
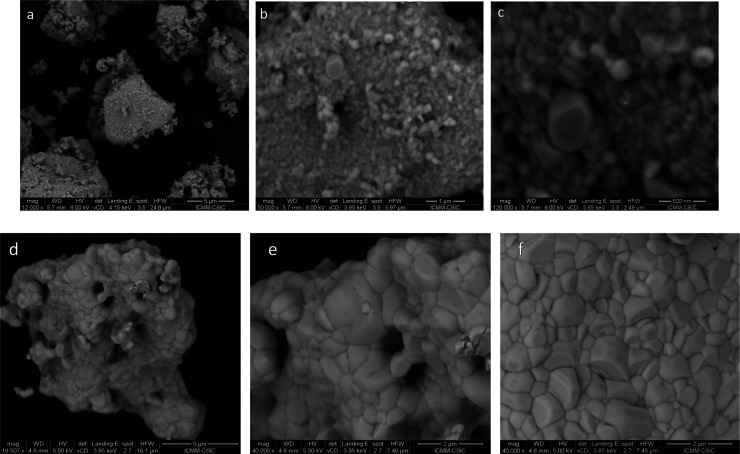
FE-SEM images with (a) 12,500×, (b) 50,000×, and (c) 120,000× magnification, for Rb_3_Bi_2_Br_9_, and (d) 18,000×, (b) 40,000×, and (c) 40,000×, for Rb_3_Bi_2_Br_6_I_9_.

### 3.2 Structural Analysis from SXRD Data

A detailed structural analysis of Rb_3_Bi_2_Br_9_ was performed using SXRD data collected from 295 up to 656 K. At room temperature, the diffraction pattern confirmed a monoclinic symmetry (space-group: *P*2_1_/*c*, standard setting, *Z* = 4). In this structure, all Rb^+^, Bi^3+^, and Br^–^ ions are located at the 4*e* (*x*, *y*, *z*) Wyckoff sites, with three distinct rubidium sites, two bismuth, and nine bromine sites. The Rietveld refinement at room temperature, from SXRD, is plotted in Figure 4a. The refined crystallographic parameters are listed in Table 1. In Figure 4b, the crystallographic structure consists of layers of [BiBr_6_] octahedra connected at their corners. Each octahedron is linked to three neighboring octahedra through shared corners. The remaining three bromine atoms, located on the opposite face of the octahedron, are coordinated with rubidium cations (Rb2 and Rb3) positioned between the layers. The excellent crystallinity of the sample, combined with the high-quality of the diffraction patterns, enabled the refinement of the anisotropic displacement parameters (ADPs) with high precision. Figure 4c provides two perspectives of a section of the unit-cell, highlighting the polyhedral connectivity and the shape of the anisotropic displacement ellipsoids.

**Figure 4:**
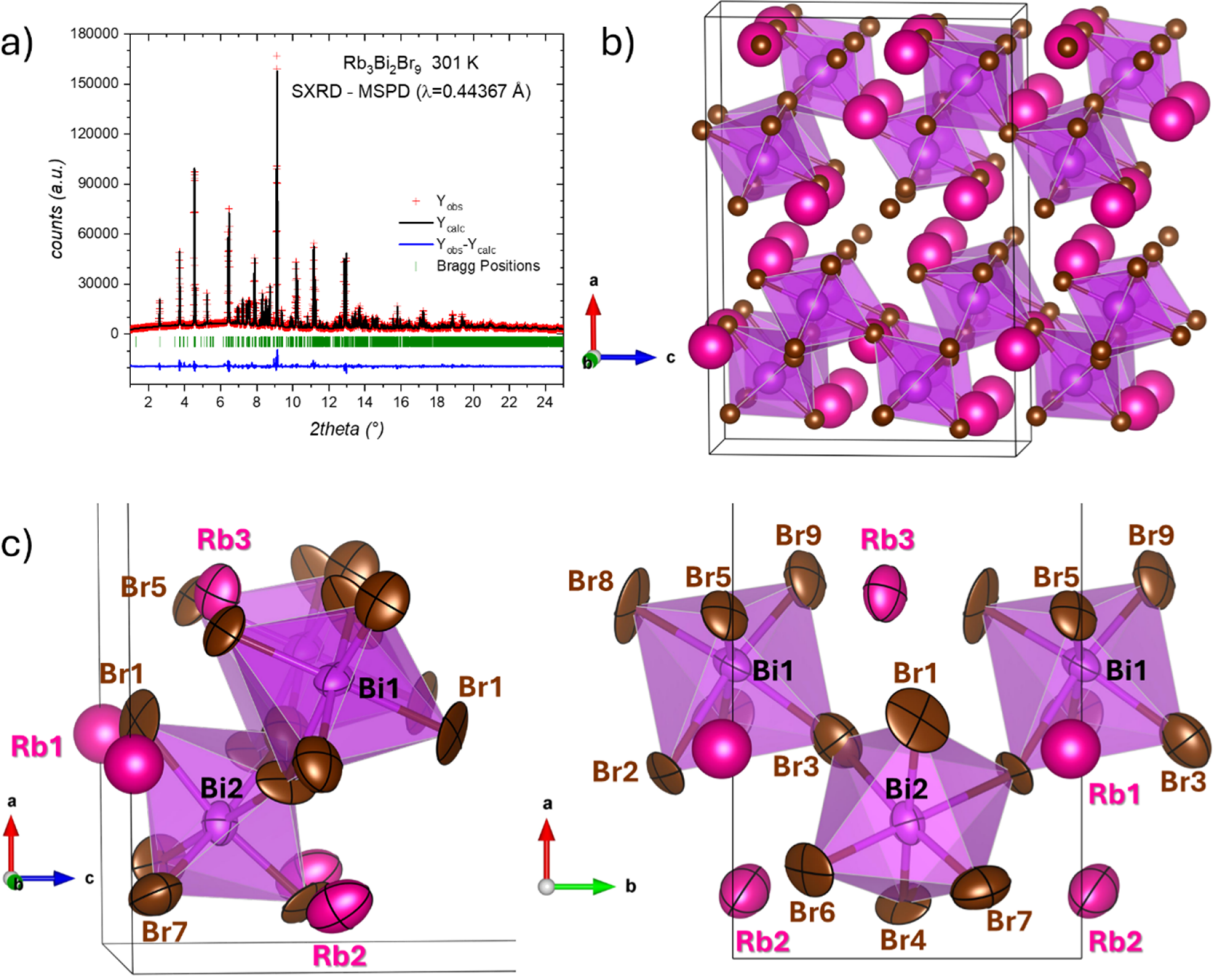
(a) Rietveld refinement of SXRD pattern, collected at room temperature. Rietveld plot: observed (red crosses) and calculated (black line) synchrotron X-ray diffraction pattern. Blue lines are the fit residuals, and the green bars are the expected Bragg reflections. (b) Schematic view of the monoclinic crystal structure. In the structural model, Rb, Bi, and Br atoms are represented by pink, purple, and bronze spheres, respectively. (c) Detailed views (along *b*- and *c*-axis) of the polyhedral connectivity and the shape of the anisotropic displacement ellipsoids (ADPs).

**Table 1:** Crystallographic Data for Rb_3_Bi_2_Br_9_ Halide from SXRD Data at 300 K, Defined in the Monoclinic *P*2_1_/*c* (Standard Setting) Space-Group, *Z* = 4. *a* = 19.4047(3) Å, *b* = 7.9112(1) Å, *c* = 13.5982 (3) Å, β = 90.5809(4)°, and *V* = 2087.41(5) Å^3^

atom	*x*	*y*	*z*	*U*_eq_ (Å^2^)	*f*_*occ*_
Rb1	0.2481(9)	0.5297(18)	0.0101(13)	0.110(4)*	1
Rb2	0.0799(5)	0.4421(15)	0.3457(8)	0.076(11)	1
Rb3	0.4281(5)	0.0459(16)	0.1795(8)	0.072(9)	1
Bi1	0.6569(2)	0.0077(8)	0.1592(3)	0.039(3)	1
Bi2	0.1578(2)	0.0066(8)	0.1730(3)	0.041(3)	1
Br1	0.2787(4)	0.008(2)	0.0381(7)	0.085(9)	1
Br2	0.2163(7)	0.7075(14)	0.2701(11)	0.081(9)	1
Br3	0.2567(7)	0.2043(15)	0.3012(11)	0.096(10)	1
Br4	0.0643(5)	0.0020(18)	0.3170(8)	0.072(8)	1
Br5	0.4001(5)	0.4929(17)	0.1600(8)	0.080(7)	1
Br6	0.1041(7)	0.2747(13)	0.0836(11)	0.066(11)	1
Br7	0.0825(7)	0.7898(13)	0.0531(10)	0.069(11)	1
Br8	0.5858(7)	0.2965(13)	0.1099(11)	0.068(13)	1
Br9	0.4418(7)	0.3086(13)	0.4160(10)	0.072(10)	1

atomic displacement parameters (Å^2^)						
	*U* ^11^	*U* ^22^	*U* ^33^	*U* ^12^	*U* ^13^	*U* ^23^
Rb1	0.142(2)	0.104(2)	0.084(3)	0.017(5)	0.001(2)	0.008(2)
Rb2	0.104(2)	0.063(4)	0.063(4)	–0.025(3)	0.027(3)	–0.020(3)
Rb3	0.063(4)	0.066(4)	0.088(2)	0.031(2)	0.016(3)	0.015(1)
Bi1	0.035(2)	0.042(1)	0.041(2)	–0.002(5)	0.0007(5)	–0.003(2)
Bi2	0.045(1)	0.034(2)	0.044(2)	–0.002(5)	0.0044(1)	–0.000(2)
Br1	0.076(5)	0.147(5)	0.033(2)	0.025(3)	0.022(3)	–0.006(2)
Br2	0.060(5)	0.091(3)	0.092(3)	0.022(5)	0.001(2)	0.036(2)
Br3	0.084(4)	0.116(3)	0.087(3)	–0.079(3)	–0.009(2)	–0.012(4)
Br4	0.088(4)	0.117(3)	0.080(3)	–0.080(3)	–0.009(2)	–0.012(4)
Br5	0.095(3)	0.111(3)	0.034(2)	0.086(3)	0.040(1)	0.030(4)
Br6	0.065(5)	0.032(3)	0.102(3)	0.022(5)	–0.007(5)	–0.016(4)
Br7	0.079(4)	0.060(5)	0.067(4)	0.012(1)	–0.016(1)	–0.051(4)
Br8	0.091(3)	0.028(2)	0.086(3)	0.018(1)	–0.021(1)	–0.036(4)
Br9	0.106(2)	0.035(3)	0.076(4)	0.01100(4)	–0.014(2)	0.015(2)
*R*_p_ = 3.03%, *R*_wp_ = 4.52%, *R*_exp_ = 1.33%, χ^2^ = 11.6, *R*_Bragg_ = 5.12%						

From the temperature-dependent SXRD data, we observed a structural phase transition at 450 K, leading to a trigonal symmetry. In Figure 5a, the thermal evolution of the main diffraction line is plotted. The high-temperature polymorph was successfully modeled in the *P* 3 *m*1 (#164) space-group, *Z* = 1. The Rietveld refinement of SXRD pattern collected at 507 K is plotted in Figure 5b. In this model, Bi cations occupy the 2d (^1^/_3_,^2^/_3_, *z*) Wyckoff site, while the bromide ions are distributed over the 3e (^1^/_2_, 0, 0) and 6i (*x*, −*x*, *z*) positions, assigned to the leveled Br1 and Br2 atoms, respectively. The rubidium cations also are distributed in two sites: 1a (0, 0, 0) and 2d (^1^/_3_,^2^/_3_, *z*) named Rb1 and Rb2, respectively. The main crystallographic results are listed in Table 2, and a schematic view of structure is represented in Figure 5c. Both high- and low-temperature polymorphs exhibit the same polyhedral arrangement, layers formed by octahedra connected to three others through adjacent corners. The key difference lies in the octahedra tilting observed in the monoclinic phase (*P*2_1_/*c*), which contrasts with the symmetric octahedra arrangement observed in the trigonal phase (*P* 3 *m*1), see in Figure S2.

**Figure 5:**
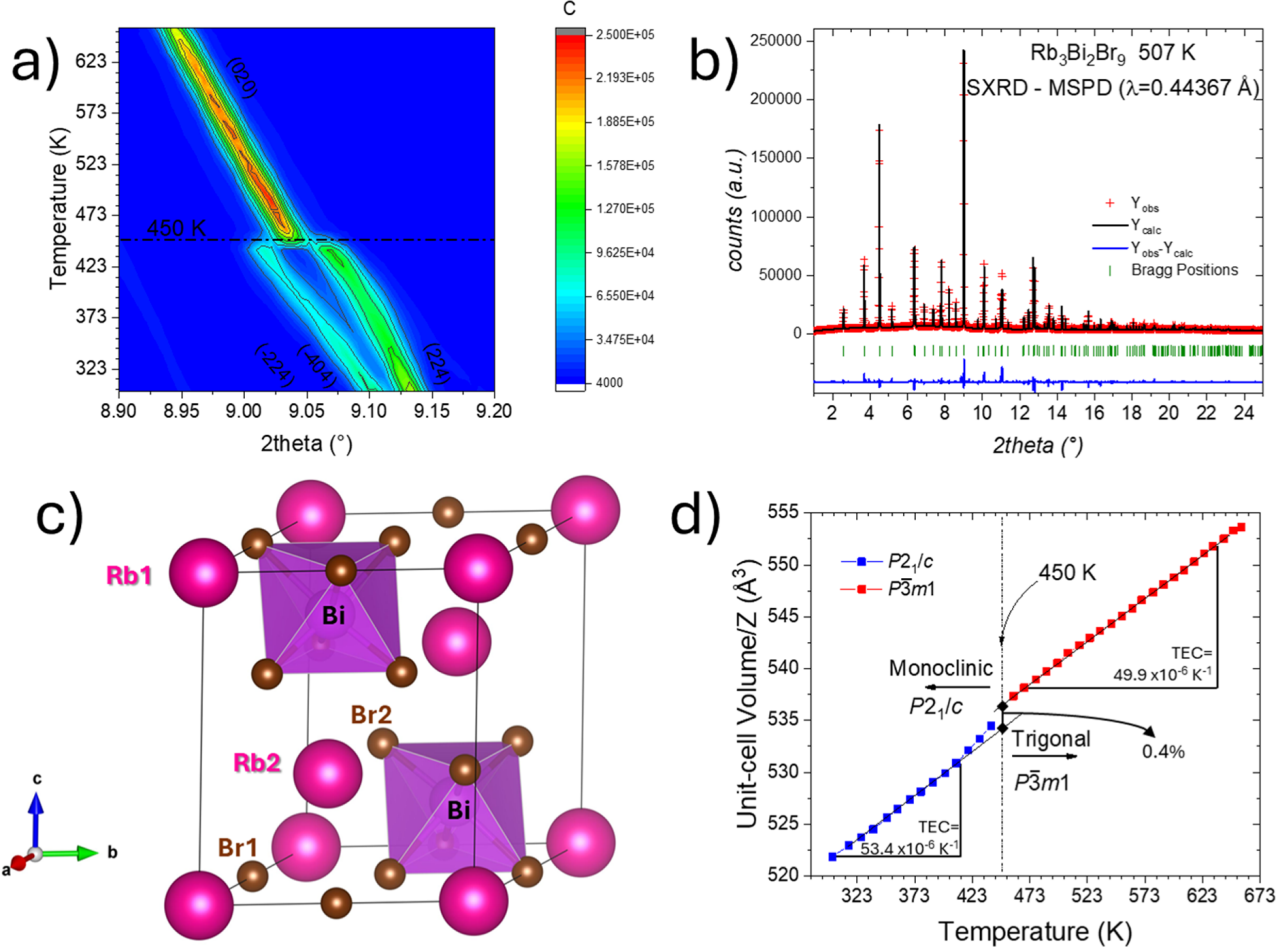
(a) A color map illustrates the temperature-dependent evolution of the main reflection in SXRD patterns collected over the 300–650 K range. (b) Rietveld refinement of SXRD pattern collected at 507 K. Rietveld plot: observed (red crosses) and calculated (black line) synchrotron X-ray diffraction pattern. Blue lines are the fit residuals, and the green bars are the expected Bragg reflections. (c) Schematic view of the trigonal crystal structure at 507 K. Rb, Bi, and Br atoms are drawn as pink, purple, and bronze spheres, respectively. (d) Thermal evolution of unit-cell volume per formula (*V*/*Z*).

**Table 2:** Crystallographic Data for Rb_3_Bi_2_Br_9_ Halide from SXRD Data at 507 K, Defined in the Trigonal *P* 3 *m*1 Space-Group, *Z* = 1. *a* = 7.9634(2) Å, *c* = 9.8424(2) Å, and *V* = 540.54(2) Å^3^

atom	*x*	*y*	*z*	*U*_eq_ (Å^2^)	*f*_occ_
Rb1	0	0	0	0.184(12)	1
Rb2	0.33333	0.66667	0.6590(8)	0.147(5)	1
Bi	0.33333	0.66667	0.1871(2)	0.0657(14)	1
Br1	0.5	0	0	0.235(7)	1
Br2	0.1716(3)	0.8284(3)	0.3345(4)	0.186(3)	1

atomic displacement parameters (Å^2^)						
	*U* ^11^	*U* ^22^	*U* ^33^	*U* ^12^	*U* ^13^	*U* ^23^
Rb1	0.129(9)	0.129(9)	0.29(2)	0.064(9)	0	0
Rb2	0.154(4)	0.154(4)	0.133(8)	0.077(4)	0	0
Bi	0.0682(15)	0.0682(15)	0.0606(14)	0.0341(15)	0.00000	0.00000
Br1	0.295(7)	0.180(6)	0.230(9)	0.090(6)	0.073(3)	0.147(3)
Br2	0.191(3)	0.191(3)	0.177(4)	0.128(3)	0.043(2)	–0.043(2)
*R*_p_ = 3.5%, *R*_wp_ = 5.70%, χ^2^ = 17.8, *R*_Bragg_ = 3.21%						

### 3.3 Structural Analysis from NPD Data

The NPD data were collected at 295 K for Rb_3_Bi_2_Br_9_ and the iodine-doped Rb_3_Bi_2_Br_6_I_3_ phases. Both patterns were well-indexed in the monoclinic *P*2_1_/*c* space-group, in accordance with synchrotron diffractions analysis. The Rietveld refinements are plotted in Figure 6; Tables S2 and S3 list the main crystallographic results for undoped and iodine-doped phase, respectively. As explained above, in this monoclinic model the bromine atoms are distributed in nine distinct crystallographic sites. Therefore, for the iodine-doped phase, there are numerous possible ways to distribute six bromines and three iodines within the structure. Furthermore, due to fitting parameter coupling, it is impossible to individually refine the Br/I ratio at each site. To address this issue, preliminary comparisons were made with the undoped phase before incorporating iodine into the refinements. Specifically, displacement factors and Bi–X (X = Br, I) bond distances were evaluated. This analysis identified selected distances (from Br4 to Br9) that were longer than those in the undoped phase, suggesting the presence of iodine at these sites. As illustrated in Figure 4c, two types of halide sites can be distinguished: the bridging positions (Br1 to Br3) and the terminal positions (Br4 to Br9). Therefore, iodine exhibits a clear preference for the terminal sites, which correspond to the halides that link one layer to the next. Based on this observation, an initial refinement model was built distributing iodine equally among these six halide sites, which resulted in a good fit of the NPD pattern. Further refinement runs were conducted to adjust the Br/I ratio at these sites; however, the results did not significantly improve the refinement. The obtained occupation factors suggest that iodine is equally distributed in the Br4 to Br9 sites. For this reason, and to avoid fluctuations in the refinements, the occupancy values were fixed at 0.5/0.5, as listed in Table S3.

**Figure 6:**
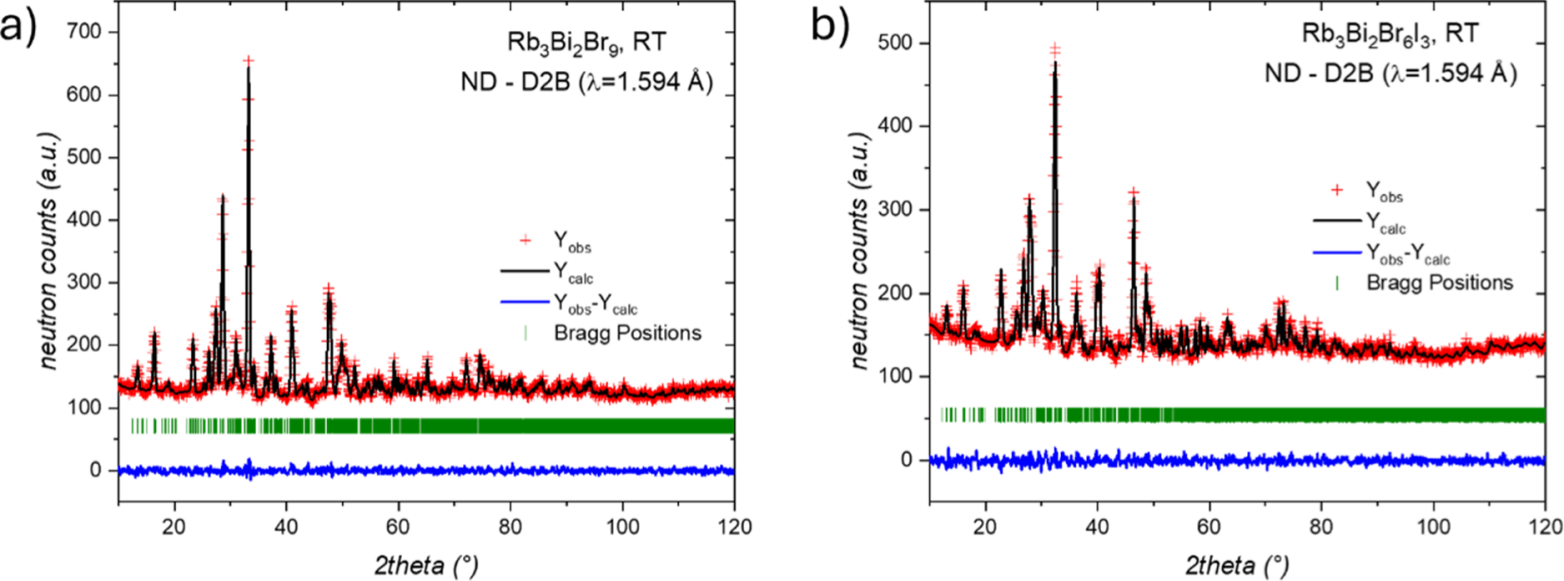
Rietveld refinement of NPD patterns collected at room temperature for (a) Rb_3_Bi_2_Br_9_ and (b) Rb_3_Bi_2_Br_6_I_3_. Rietveld plot: observed (red crosses) and calculated (black line) neutron diffraction pattern. Blue lines are the fit residuals, and the green bars are the expected Bragg reflections.

### 3.4 Optical Properties

The optical properties of Rb_3_Bi_2_Br_9_ and Rb_3_Bi_2_Br_6_I_3_ powders were examined using diffuse reflectance UV–vis spectroscopy. The optical absorption coefficient derived from the Kubelka–Munk function is presented in Figure 7a, *F*(*R*)=(1 −*R*)^2^/2*R*, where *R* represents the reflectance of each sample, plotted against the wavelength in units of eV. The direct bandgap of each perovskite was determined by extrapolating the linear region of the absorption edge to the *x*-axis. Rb_3_Bi_2_Br_9_ exhibited a bandgap of ∼2.70 eV. As anticipated, a red shift was observed for Rb_3_Bi_2_Br_6_I_3_, resulting in a reduced bandgap of ∼2.20 eV. This shift is consistent with the substitution of bromine by iodine, which effectively narrows the bandgap, enhancing the material’s potential for optoelectronic applications.^52,67^ In addition, this all-inorganic halide family arises as a promising alternative to the hybrid halide family, such as MA_3_Bi_2_Br_9–*x*_I_*x*_. The band gap evolution with increasing iodine content for both Rb and MA phases is illustrated in Figure 7b. Hence, the present all-inorganic phases are members of a more stable series, offering the potential to tune the bandgap within a similar range as the less stable hybrid halide phases. This enhanced stability, combined with comparable tunability, makes the all-inorganic halide family a compelling candidate for applications requiring robust materials with adjustable optical and electronic properties.

**Figure 7:**
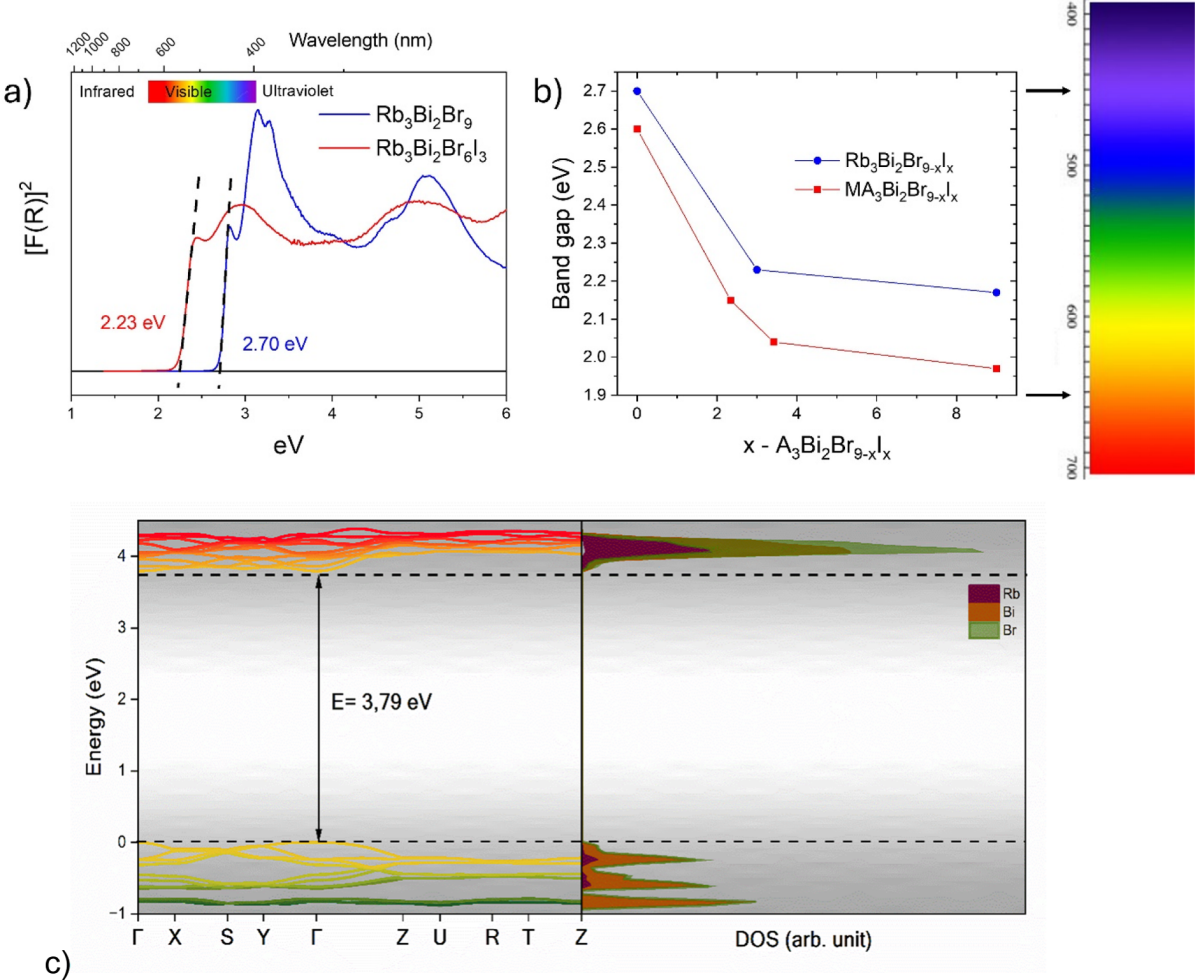
(a) Room temperature Kubelka–Munk transformed diffuse reflectance spectra of Rb_3_Bi_2_Br_9_ and Rb_3_Bi_2_Br_6_I_3_. (b) Comparison of the optical band gap energy for Rb_3_Bi_2_Br_9–*x*_I_*x*_ and MA_3_Bi_2_Br_9–*x*_I_*x*_. (c) Electronic properties of the Rb_3_Bi_2_Br_9_ system obtained via DFT calculations, showing the estimated values for the band structure (right) and the density of states—DOS (left).

According to the theoretical band structure, the bulk model for Rb_3_Bi_2_Br_9_ showed an estimated direct band gap value of ∼3.79 eV, along the Γ–Γ path in the first Brillouin zone. Although this value is higher than the experimentally obtained one (∼2.7 eV), the band gap can be considered satisfactory, considering the low symmetry of the space-group and factors such as the choice of functionals. Regarding the projected density of states, the distribution of the occupied electronic states exhibits a greater contribution from Br and Bi orbitals in the valence band, with relatively similar occupation levels between both atoms. In contrast, the conduction band is mainly composed of bromide orbitals, with a lower contribution from bismuth orbitals in the electronic transition processes. This is due to the Br orbitals being more prone to absorb photon excitation energy, because of their higher electronegativity.

## 4 Discussion

### 4.1 Thermal Expansion

The thermal evolution of the unit-cell volume (normalized by *Z*) is represented in Figure 5d. Aside from the region near the structural phase transition, the average thermal expansion remains relatively constant below and above ∼450 K. The thermal expansion coefficients (TEC) estimated via linear extrapolation of the volume vs temperature curve are 53.4 ppm·K^–1^ and 49.9 ppm·K^–1^ for monoclinic and trigonal phases, respectively. The phase transition results in an increase in volume of approximately 0.4%. The thermal evolution of specific structural distances within the layered arrangement, such as layer spacing, width, and length, was analyzed, as plotted in Figures S3 and S4. These plots reveal that the expansion is not monotonic as the temperature increases, as indicated by the variation in unit-cell volume. The interlayer spacing expansion exhibits discontinuity at the phase transitions, with a lower TEC in the trigonal phase compared to the monoclinic phase (see Figure S3). Regarding the intralayer distances, two characteristic distances can be distinguished: length and width, as defined in Figure S4. On the contrary, the layer length depicts a clear discontinuity at the phase transitions, with a significant decrease in the TEC. In contrast, the layer width remains unchanged in the monoclinic phase, but after the transition, it increases in the same way that layer length, which is consistent with the trigonal symmetry. Both intralayer behaviors can be explained in terms of [BiBr_6_] octahedra distortion. This distortion can be described by two types of octahedral tilting illustrated in Figure S2a,b, which account for the variations in width (Figure S4b) and length (Figure S4a), respectively. Furthermore, the octahedral tilting also affects the β angle of the unit-cell, as shown in Figure S3b. This angle represents the relationship between the layer length and the layer spacing, indicating that the layers are slightly displaced along *c* direction in the monoclinic symmetry. The temperature-dependent structural changes further support the laminar character of the phase and the nature of its chemical bonding. Indeed, as discussed in Table S5 from topochemical analysis using DFT, the bonding framework in Rb_3_Bi_2_Br_9_ consists of a combination of ionic (Rb–Br) and partially covalent (Bi–Br) interactions, i.e. an archetypal bonding scheme for halide perovskite structures.

### 4.2 Mechanism of Phase Transition

To shed light into the mechanism of the structural transition in Rb_3_Bi_2_Br_9_, we employed symmetry mode analysis (or distortion mode analysis). The amplitudes and polarization vectors of the symmetry-adapted distortion modes were calculated for the low-symmetry phase (subgroup *H*: here, monoclinic *P*2_1_/*c* space-group from SXRD structural refinement at 300 K), with respect to its high-symmetry counterpart (supergroup *G*: here, trigonal *P* 3 *m*1 space-group from SXRD structural refinement at 656 K), using the *Amplimodes* algorithm available at the Bilbao Crystallographic Server.^68–70^ The low-symmetry distorted unit-cell can be expressed as a superposition of “frozen” distortion modes, each transforming according to an irreducible representation (irrep) of the high-symmetry space-group. The starting point of the analysis involves expressing the atomic positions of the low-symmetry phase (***r***; subgroup *H*) in terms of those of the high-symmetry structure (***r***_0_; supergroup *G*), with the latter transformed into the unit-cell basis of the subgroup *H*, as given by:

1 $$u\left(\mu ,i\right)=r\left(\mu ,i\right)-r_{0}\left(\mu ,i\right)=\sum_{\tau ,m}A_{\tau ,m}{ê}_{\tau ,m}\left(\mu ,i\right)$$

where μ denotes the distinct atomic species (or crystallographic sites) in the asymmetric unit, and *i* = 1··· *n*_μ_ indexes the atoms associated with each site. To describe the structural evolution, the displacement vectors ***u*** (μ,*i*) are linearly decomposed onto the basis vectors of the irreducible representations (*irreps*) derived from the group-subgroup analysis. Here, ${ê}_{\tau ,m}\left(\mu ,i\right)$ denotes the polarization (basis) vector of the irreducible representation τ, with *m* indexing the independent modes associated with each τ, and *A*_τ,*m*_ stands for the corresponding distortion amplitude.

To perform the symmetry-mode decomposition, we used the experimentally determined structures, described in the *P*2_1_/*c* at 300 K (subgroup *H*) and *P* 3 *m*1 at 656 K (supergroup *G*) space-groups. The irreps are labeled using the *k*-vector designation in the first Brillouin-zone, viz. Γ, *A*, *L*, and *M*. The decomposition of the low-symmetry phases in terms of the irreducible representations of the high-symmetry supergroup *G* results in the Wyckoff site splitting listed in Table 3. The corresponding symmetrical mode amplitudes are summarized in Table 4. Based on this site-splitting framework, the number and symmetry characteristics of the distortion modes can be determined for each distorted phase.

**Table 3:** Symmetry Mode Analysis of the Rb_3_Bi_2_Br_9_ Structure Showing the Wyckoff Site Splitting for the Low Symmetry Phase (*P*2_1_/*c* at 300 K) and the Symmetry-Adapted Modes Responsible for the Symmetry Lowering from the High Symmetry Phase (*P* 3 *m*1 at 656 K)

supergroup *G*	subgroup *H*	symmetry-adapted modes
*P* 3 *m*1 (#164)	*P*2_1_/*c* (#14)	
Rb1 1a	Rb1 4e	*A*_2_^–^(1) + *A*_3_^–^(1) + *L*_1_^–^(1)
Rb2 2d	Rb2–3 4e	Γ_1_^+^(1) + Γ_3_^+^(1)+ *A*_2_^–^(1) + *A*_3_^–^(1) + *L*_1_^–^(1) + *M*_2_^+^(1)
Bi1 2d	Bi1–2 4e	Γ_1_^+^(1) + Γ_3_^+^(1)+ *A*_2_^–^(1) + *A*_3_^–^(1) + *L*_1_^–^(1) + *M*_2_^+^(1)
Br1 3e	Br1–3 4e	*A*_2_^–^(2) + *A*_3_^–^(3) + *L*_1_^–^(1) + *M*_2_^+^(3)
Br2 6i	Br4–9 4e	Γ_1_^+^(2) + Γ_3_^+^(3)+ *A*_2_^–^(2) + *A*_3_^–^(3) + *L*_1_^–^(4) + *M*_2_^+^(4)

**Table 4:** Amplitudes of the Symmetry-Adapted Modes as Normalized within the Primitive Unit-Cell of the High Symmetry Structure (*P* 3 *m*1, Reference Structure at 656 K in Table S1) with Their Respective Direction and Dimension (Multiplicity) for the Monoclinic Phase

Irreps	Γ_1_^+^	Γ_3_^+^	*A*_2_^–^	*A*_3_^–^	*L*_1_^–^	*M*_2_^+^
direction	(a)	( $-\frac{1}{2}a,\frac{\sqrt{3}}{2}a$ )	(a)	( $-\frac{\sqrt{3}}{2}a,-\frac{1}{2}a$ )	(0 a 0)	(0 a 0)
dimension	4	5	7	9	8	9
amplitude (Å)	0.1257	0.0786	0.1390	1.4038	0.6654	1.2380
*k*-vector	(0,0,0)	(0,0,0)	( $0,0,\frac{1}{2}$ )	( $0,0,\frac{1}{2}$ )	( $\frac{1}{2},0,\frac{1}{2}$ )	( $\frac{1}{2},0,0$ )
isotropy subgroup	*P*3̅̅*m*1	*C*2/*m*	*P*3̅̅*m*1	*C*2/*m*	*C*2/*c*	*P*2_1_/*c*
						

In the monoclinic structure, forty-two distortion modes as written by six irreps, viz Γ_1_^+^, Γ_3_^+^, *A*_2_^–^, *A*_3_^–^, *L*_1_^–^, and *M*_2_^+^ are allowed, as shown in Table 3. Rb1 site at 1a enables three modes [*A*_2_^–^(1) + *A*_3_^–^(1) + *L*_1_^–^(1)] at Rb1 site 4*e* in the monoclinic unit-cell. Rb2 sites at 2d split into two sites Rb2 and Rb3, which leads to six distortion modes [Γ_1_^+^(1) + Γ_3_^+^(1)+ *A*_2_^–^(1) + *A*_3_^–^(1) + *L*_1_^–^(1) + *M*_2_^+^(1)]. At Bi1 sites (2d), a splitting is also observed for stabilizing Bi1 and Bi2 at 4e in monoclinic unit-cell, and consequently generating six distortion modes [Γ_1_^+^(1) + Γ_3_^+^(1)+ *A*_2_^–^(1) + *A*_3_^–^(1) + *L*_1_^–^(1) + *M*_2_^+^(1)]. At Br1 sites (3e), the site splitting induces three Br positions (Br1, Br2, and Br3) as driven by nine distortion modes, namely: *A*_2_^–^(2) + *A*_3_^–^(3) + *L*_1_^–^(1) + *M*_2_^+^(3). At Br2 sites (6i), the splitting leads more degree of freedom along six Br positions (Br4, Br5, Br6, Br7, Br8, and Br9) as induced by 18 distortion modes [Γ_1_^+^(2) + Γ_3_^+^(3)+ *A*_2_^–^(2) + *A*_3_^–^(3) + *L*_1_^–^(4) + *M*_2_^+^(4)].

From the symmetry mode amplitudes listed in Table 4, we observed that the monoclinic distortion is principally induced by the modes *M*_2_^+^ (amplitude of ∼1.238 Å) and *A*_3_^–^ (amplitude of ∼1.404 Å), that resembles rigid rotations of neighboring [BiBr_6_] octahedra along the *a*- and *b*-axes, respectively, see the polarization vectors illustrated in Figure 8. The distortion modes Γ_1_^+^ and Γ_3_^+^ correspond to octahedral stretching and bending, respectively, but exhibit low amplitudes of ∼0.126 and ∼0.079 Å. As a result, they contribute less significantly to the overall monoclinic distortion. The remaining distortion modes, *A*_2_^–^ (∼0.139 Å) and *L*_1_^–^ (∼0.665 Å), correspond to rigid translations of Rb atoms along the *a*- and *b*-axes, respectively, and contribute moderately to the overall monoclinic distortion.

**Figure 8:**
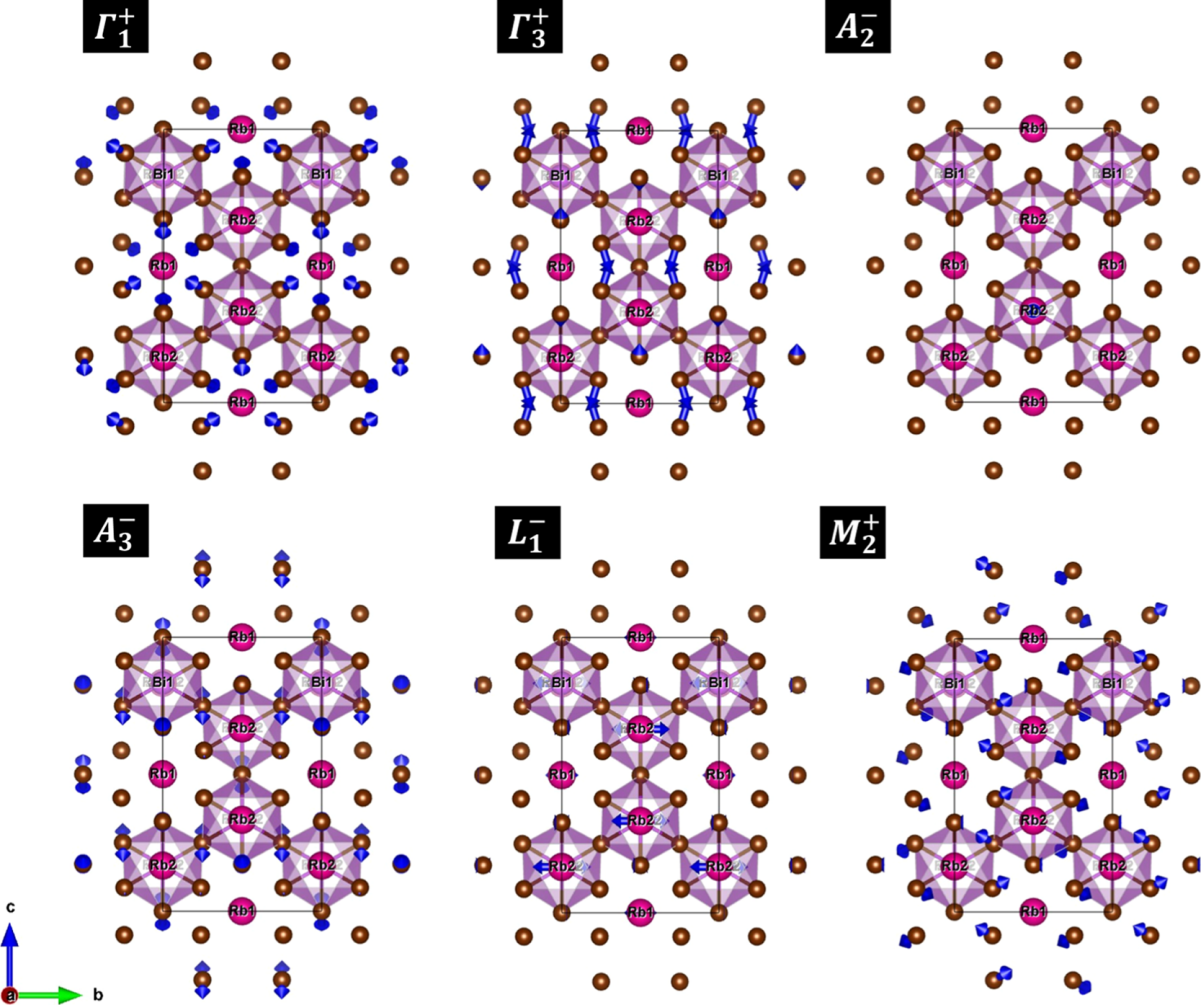
Schematic illustrations of atomic displacements (polarization vectors) corresponding to symmetry-adapted modes involved in the symmetry-lowering transition $P3̅m1\to P2_{1}/c$ . Blue arrows indicate the amplitudes and directions of the symmetry-adapted distortions as calculated using *Amplimodes* algorithm.

## 5 Conclusions

This work presents a thorough investigation of the structural and optoelectronic properties of lead-free Rb_3_Bi_2_Br_9_ and Rb_3_Bi_2_Br_6_I_3_ halides, synthesized through a solvent-free mechanochemical route. The resulting materials exhibit excellent crystallinity and phase purity, as confirmed by synchrotron and neutron powder diffraction data. Both compounds adopt a monoclinic layered structure at room temperature, composed of [BiBr_6_] octahedra connected in a 2D framework. Upon heating, a first-order structural phase transition to a high-symmetry trigonal phase occurs near ∼450 K, without decomposition, demonstrating good thermal stability and reversibility. Detailed symmetry-adapted distortion mode analysis revealed that the monoclinic-to-trigonal transformation is primarily driven by octahedral rotations and tilting within the [BiBr_6_] framework. These distortions dominate the symmetry breaking, while octahedral stretching, bending, and rigid Rb translations play secondary roles. The precise quantification of these distortion modes offers a clear mechanistic understanding of the transition and highlights the structural flexibility of these layered perovskite derivatives. Optical studies indicate a direct bandgap of ∼2.70 eV for Rb_3_Bi_2_Br_9_ and ∼2.21 eV for the iodine-substituted Rb_3_Bi_2_Br_6_I_3_, confirming that halide substitution effectively tunes the optical response. This tunability, coupled with theoretical support from DFT calculations, reinforces the potential of Rb-based A_3_B_2_X_9_ halides as stable, lead-free alternatives for optoelectronic industrial applications. These findings pave the way for further exploration of the Rb–Bi–halide compositional space, particularly for applications in photovoltaics, photodetectors, and other light-harvesting devices where stability and environmental safety are paramount.

## Supplementary Material



## Notes

SEM images and EDX spectra; crystallographic Tables and unit-cell parameters variation; details on the topochemical analysis (PDF)
