# Supplementary material for: Structural Phase Transition and the Effect of Iodine on Phase Stability in Rb3Bi2Br9 Perovskite-Related Halides with 2D Dimensionality
Source: Inorg Chem. 2025 Oct 21;64(43):21620–31. doi: 10.1021/acs.inorgchem.5c03701 (PMC12587400; doi:10.1021/acs.inorgchem.5c03701)
Supplement: Supplementary file 1 [file ic5c03701_si_001.pdf]

SUPLEMMENTARY INFORMATION

Structural Phase Transition and the Effect of Iodine on Phase Stability in $Rb_3Bi_2Br_9$ Perovskite-Related Halides with 2D Dimensionality

Yousra Chakroun[1,2], Wajdi Cherif[3], Carlos A. López[1,4], Brenda Martinelli[5], Francielen S.M. Rodrigues[5], Federico Serrano-Sánchez[1], Javier Gainza[1,6], Romualdo S. Silva Jr.[1,7], Mateus M. Ferrer[5], José Luis Martinez[1], Maria Teresa Fernández-Díaz,[7] João Elias F.S. Rodrigues[1,6,*], and José Antonio Alonso[1,*]

Affiliation

(1) Instituto de Ciencia de Materiales de Madrid, CSIC, Cantoblanco 28049 Madrid, Spain.

(2) Laboratory Inorganic Chemistry, Faculty of Sciences of Sfax, University of Sfax 3000 Sfax, Tunisia.

(3) National Engineering School of Sfax (ENIS), Laboratory of Electromechanical Systems (LASEM), B.P.W. 3038, Sfax, Tunisia.

(4) Instituto de Investigaciones en Tecnología Química (UNSL-CONICET) and Facultad de Química, Bioquímica y Farmacia, Almirante Brown 1455 (5700) San Luis, Argentina.

(5) CCAF, PPGCEM/CDTec, Federal University of Pelotas, 96010-610 Pelotas, Rio Grande do Sul, Brazil.

(6) European Synchrotron Radiation Facility (ESRF), 71 Avenue des Martyrs, 38000 Grenoble, France.

(7) Institut Laue Langevin, BP 156X, Grenoble, F-38042, France

*** Corresponding author**

ja.alonso@icmm.csic.es (JAA); rodrigues.joaoelias@gmail.com (JEFSR).

Chemical analysis

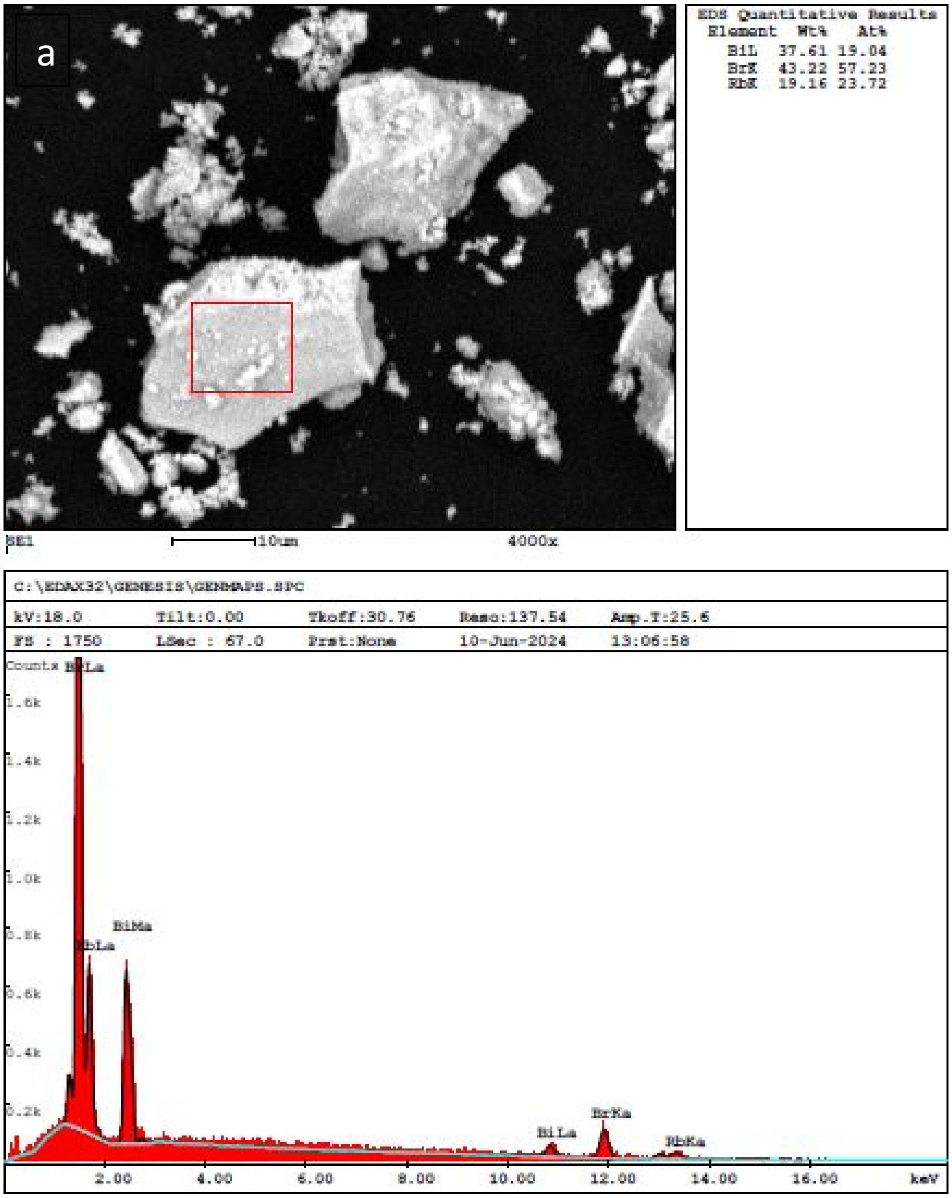

EDX for $Rb_3Bi_2Br_9$

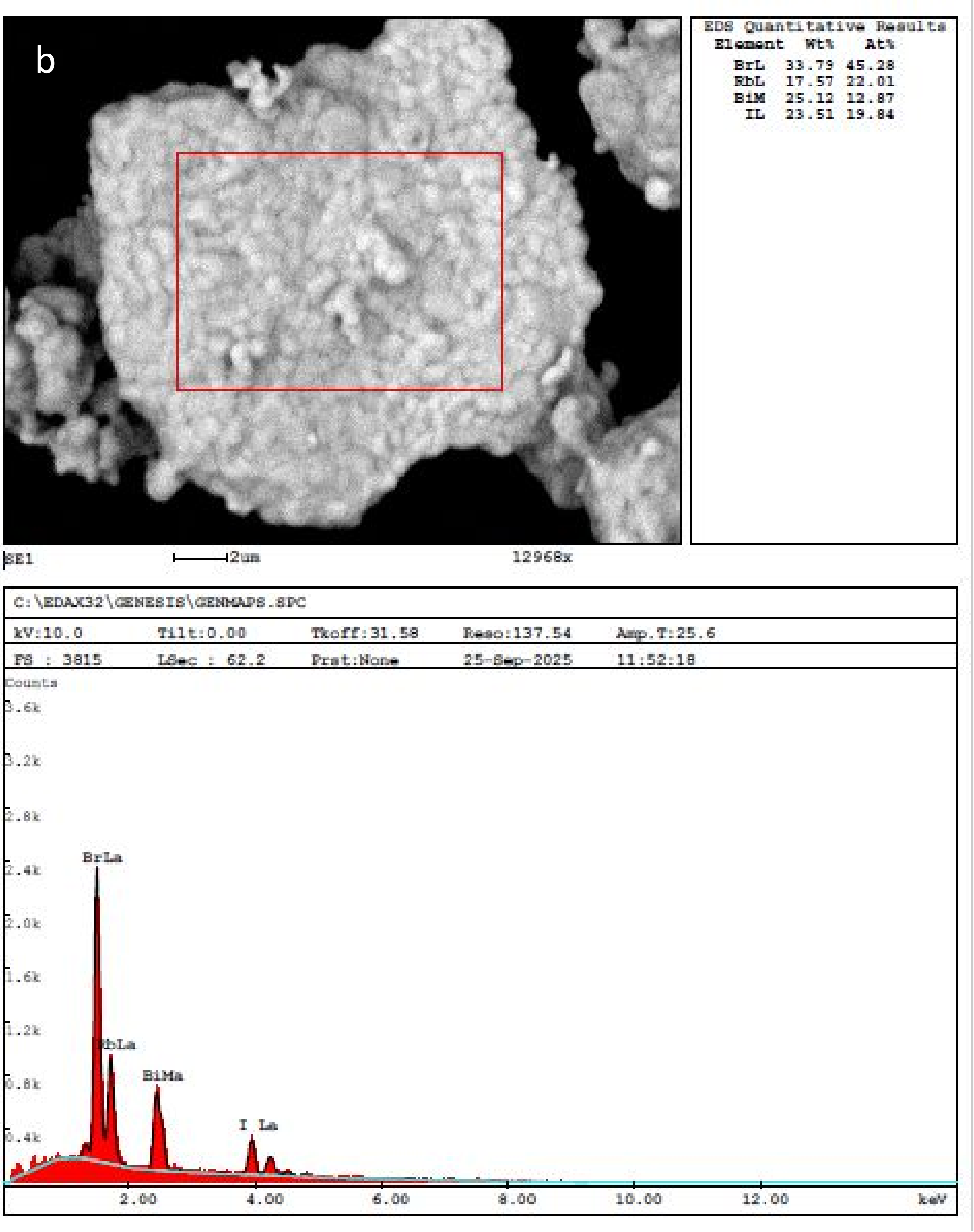

EDX for $Rb_3Bi_2Br_6I_3$

Figure S1. Chemical analysis for a) $Rb_3Bi_2Br_9$ and b) $Rb_3Bi_2Br_6I_3$. Upper panels: SEM images where the EDX spectra were collected, and relative contents of Rb, Bi and Br (I), in reasonable agreement with the expected composition. Lower panels: typical EDX spectra.

Synchrotron X-ray diffraction results

Table S1. Crystallographic data for $Rb_3Bi_2Br_9$ halide from SXRD data at 656 K, defined in the trigonal $P\bar{3}m1$ space-group. a = 8.03449(20) Å, c = 9.9032(2) Å, and V = 553.63(2) Å^3.

**Atom**	***x***	***y***	***z***	U_{iso} **(Å^2)**	f_{occ}
Rb1	0	0	0	0.273(19)	1
Rb2	0.33333	0.66667	0.6545(9)	0.183(7)	1
Bi	0.33333	0.66667	0.1861(3)	0.0883(20)	1
Br1	0.5	0	0	0.260(10)	1
Br2	0.1728(3)	0.8272(3)	0.3316(5)	0.238(4)	1

Atomic Displacement Parameters (Å^2)

	U^{11}	U^{22}	U^{33}	U^{12}	U^{13}	U^{23}
Rb1	0.143(12)	0.143(12)	0.53(3)	0.072(12)	0.00000	0.00000
Rb2	0.206(6)	0.206(6)	0.136(10)	0.103(6)	0.00000	0.00000
Bi	0.091(2)	0.091(2)	0.0822(19)	0.046(2)	0.00000	0.00000
Br1	0.305(9)	0.224(8)	0.252(12)	0.112(8)	0.055(4)	0.109(4)
Br2	0.246(4)	0.246(4)	0.222(5)	0.162(4)	0.045(3)	-0.045(3)

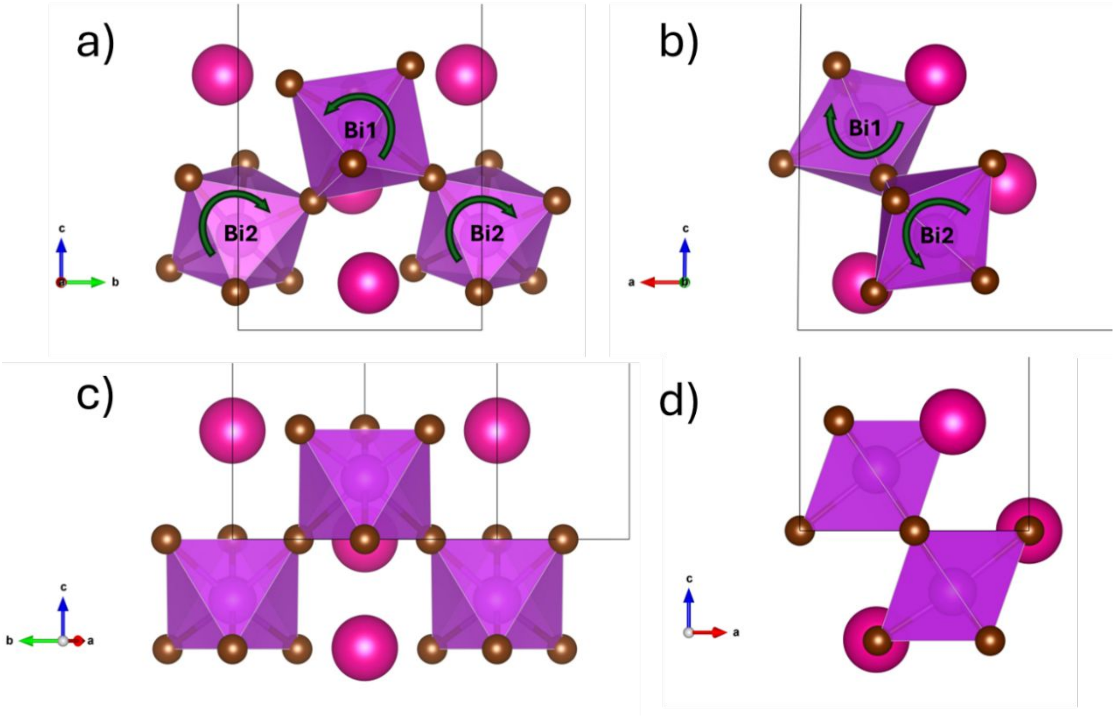

Figure S2. Schematic representation of the octahedral tilting in the monoclinic phase (a) and (b) in comparison with the trigonal phase (c) and (d).

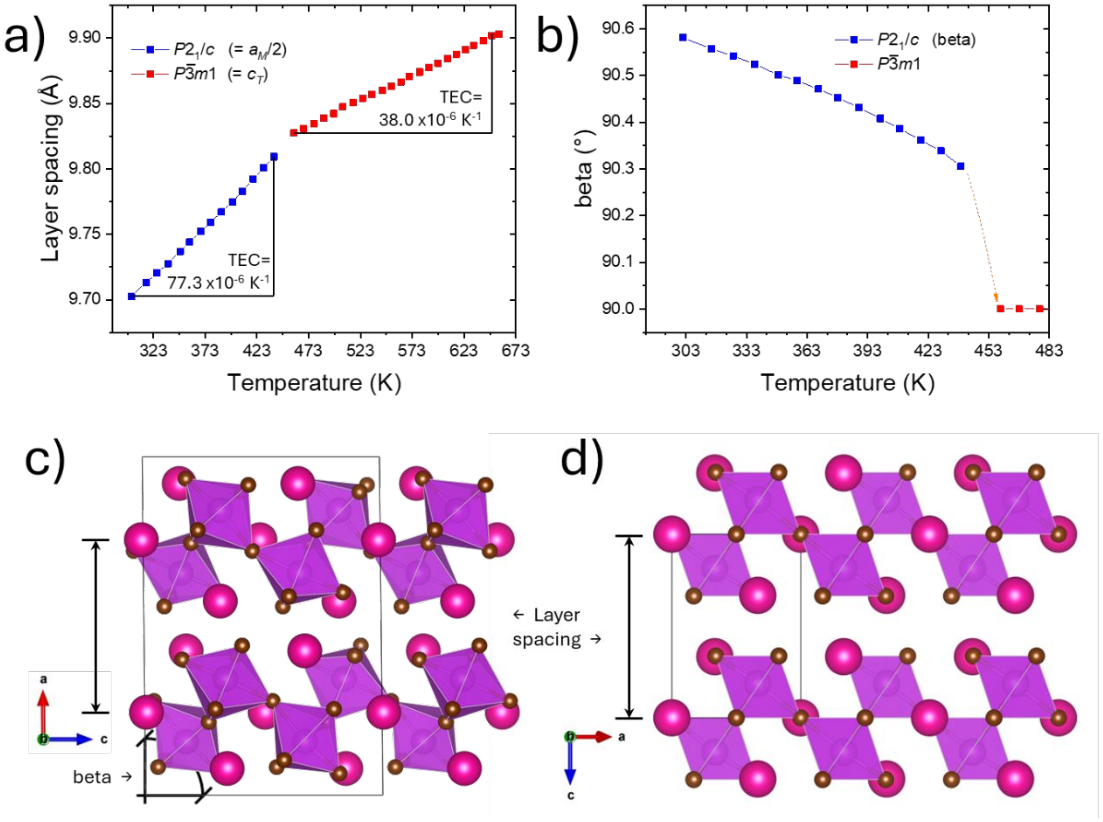

Figure S3. Thermal evolution of (a) layer spacing and (b) β angle. Schematic representation of the layer spacing in both (c) monoclinic and (d) trigonal crystal structure.

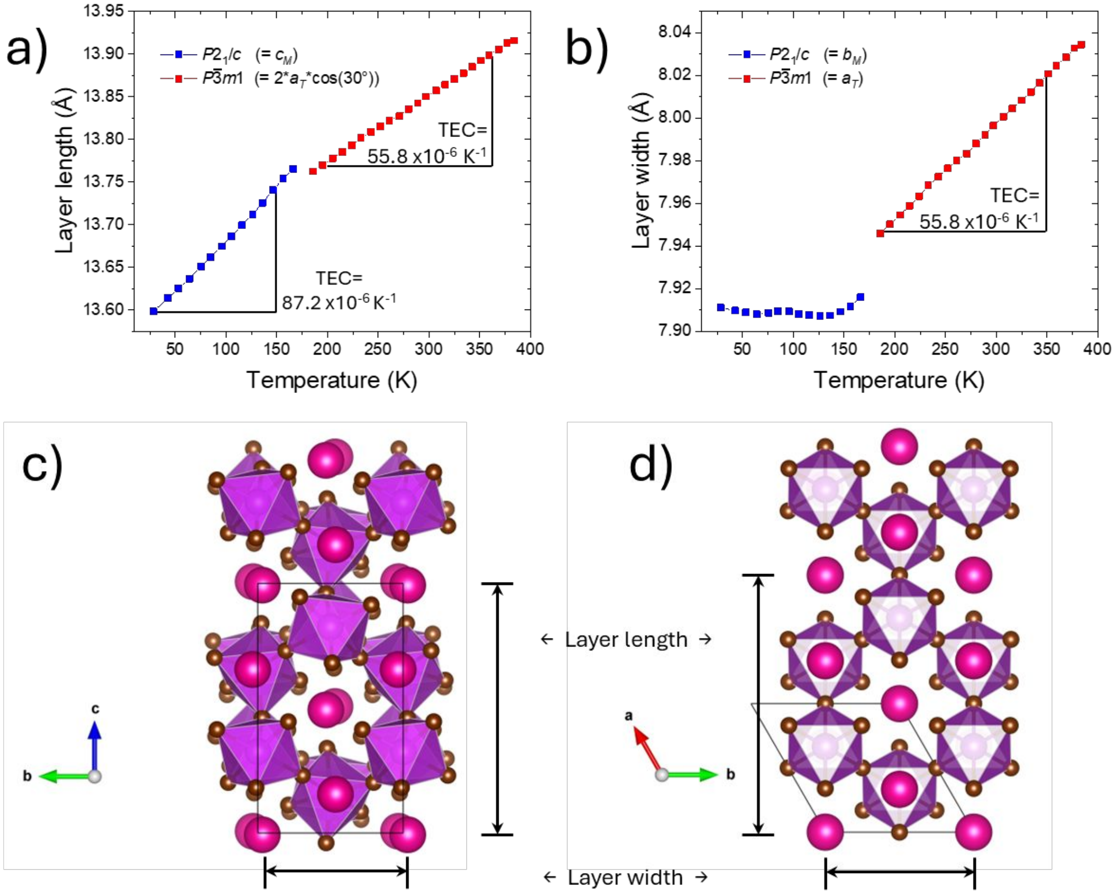

Figure S4. Thermal evolution of (a) layer length and (b) layer width. Schematic representation of the layer length and width in both (c) monoclinic and (d) trigonal crystal structures.

Neutron diffraction results

Table S2. Crystallographic data for $Rb_3Bi_2Br_9$ halide from NPD data at room temperature, defined in the monoclinic $P2_1/c$ space-group (Z = 4). a = 19.389(2) Å, b = 7.9090(7) Å, c = 13.582(1) Å, β = 90.584(5)°, and V = 2082.7(3) Å^3.

**Atom**	***x***	***y***	***z***	U_{iso} (Å^2)	f_{occ}
Rb1	0.747(2)	−0.036(3)	−0.003(3)	0.097(8)	1
Rb2	0.915(2)	0.055(5)	0.657(2)	0.11(1)	1
Rb3	0.425(1)	−0.046(3)	0.680(2)	0.066(8)	1
Bi1	0.3438(8)	0.004(2)	0.341(1)	0.035(4)	1
Bi2	0.8406(8)	0.002(3)	0.324(1)	0.032(4)	1
Br1	0.283(1)	0.034(3)	0.539(1)	0.054(6)	1
Br2	0.2143(9)	0.194(3)	0.270(2)	0.046(6)	1
Br3	0.747(1)	0.299(4)	0.700(2)	0.073(7)	1
Br4	0.061(1)	−0.002(4)	0.820(2)	0.066(6)	1
Br5	0.602(1)	−0.020(3)	0.847(2)	0.063(6)	1
Br6	0.100(2)	0.777(4)	0.086(2)	0.09(1)	1
Br7	0.917(1)	0.716(2)	0.944(1)	0.039(5)	1
Br8	0.593 (1)	0.801(3)	0.109(2)	0.058(6)	1
Br9	0.442(1)	0.704(3)	0.915(2)	0.047(6)	1

R_p = 2.24%, R_{wp} = 2.82%, R_{exp} = 1.23%, χ^2 = 5.26, R_{Bragg} = 9.19%

Table S3. Crystallographic data for $Rb_3Bi_2Br_6I_3$ halide from NPD data at room temperature, defined in the monoclinic $P2_1/c$ space-group (Z = 4). a = 19.648(4) Å, b = 8.138(1) Å, c = 13.961(3) Å, β = 90.959(9)°, and V = 2232.1(7) Å^3.

Atom	*x*	*y*	*z*	U_{iso} (Å^2)	f_{occ}
Rb1	0.756(4)	−0.039(5)	0.000(6)	0.09(2)	1
Rb2	0.906(2)	0.047(4)	0.657(3)	0.06(1)	1
Rb3	0.415(3)	−0.060(5)	0.656(4)	0.10(2)	1
Bi1	0.335(1)	−0.002(3)	0.337(2)	0.006(5)	1
Bi2	0.8370(2)	0.005(3)	0.324(2)	0.029(7)	1
Br1	0.276(2)	0.030(4)	0.521(3)	0.035(9)	1
Br2	0.221(2)	0.200(5)	0.256(2)	0.03(1)	1
Br3	0.747(3)	0.282(7)	0.715(4)	0.11(2)	1
Br4/I4	0.061(2)	−0.033(5)	0.814(4)	0.05(1)	0.5/0.5
Br5/I5	0.605(2)	−0.039(4)	0.860(3)	0.05(1)	0.5/0.5
Br6/I6	0.116(2)	0.820(5)	0.073(3)	0.04(1)	0.5/0.5
Br7/I7	0.920(2)	0.697(4)	0.947(3)	0.02(1)	0.5/0.5
Br8/I8	0.592(2)	0.784(4)	0.102(3)	0.03(1)	0.5/0.5
Br9/I9	0.446(3)	0.713(6)	0.904(3)	0.06(2)	0.5/0.5

R_p = 2.29%, R_{wp} = 2.89%, R_{exp} = 1.20%, χ^2 = 5.76, R_{Bragg} = 6.26%

Table S4. Main atomic distances for $Rb_3Bi_2Br_9$ and $Rb_3Bi_2Br_6I_3$ at 298 K extracted from NPD data.

Distances	$Rb_3Bi_2Br_9$ X (=Br)	Distances	$Rb_3Bi_2Br_6I_3$ X (=Br/I)	Increase distances (%)
Bi1–Br1	2.96(3)	Bi1–Br1	2.84(5)	-4.05
Bi1–Br2	3.07(3)	Bi1–Br2	2.99(5)	-2.61
Bi1–Br3	3.02(4)	Bi1–Br3	2.88(7)	-4.64
Bi1–Br5	2.76(3)	Bi1–Br5/I5	3.03(5)	9.78
Bi1–Br8	2.73(3)	Bi1–Br8/I8	2.92(6)	6.96
Bi1–Br9	2.70(3)	Bi1–Br9/I9	2.86(5)	5.93
<Bi1–Br>	2.8737	<Bi1–Br/I>	2.9193	1.59
Bi2–Br1	3.07(3)	Bi2–Br1	3.13(5)	1.95
Bi2–Br2	2.94(4)	Bi2–Br2	2.94(5)	0
Bi2–Br3	2.92(4)	Bi2–Br3	2.89(7)	-1.03
Bi2–Br4	2.74(3)	Bi2–Br4/I4	2.81(6)	2.55
Bi2–Br6	2.75(4)	Bi2–Br6/I6	3.07(5)	11.64
Bi2–Br7	2.78(3)	Bi2–Br7/I7	2.87(5)	3.24
<Bi2–Br>	2.8683	<Bi2–Br/I>	2.954	2.99
<Rb1–Br>	3.7102	<Rb1–Br/I >	3.9153	5.53
<Rb2–Br>	3.7302	<Rb2–Br/I >	3.8200	2.41
<Rb3–Br>	3.7986	<Rb3–Br/I >	3.8320	0.88

Topochemical analysis

The topological parameters listed in Table S5 elucidates that Rb–Br bonds are mainly ionic. This is supported by low electron density values, positive total energy density ($H > 0$), and $|v|/G$ ratios below 1, which are characteristic of interactions driven by classical effects with minimal electron sharing. The long bond lengths are also consistent with electrostatic interactions between ions. On the other hand, Bi–Br bonds are primarily covalent, with significantly higher electron density, negative H values, and $|v|/G$ ratios above 1. These findings suggest a greater quantum contribution and a tendency toward electron sharing, although it remains limited. The shorter bond lengths indicate closer proximity and increased structural rigidity of the $[BiBr_6]^{3-}$ octahedra. Therefore, the structure of $Rb_3Bi_2Br_9$ is supported by a combination of ionic (Rb–Br) and partial covalent (Bi–Br) interactions, which is a typical pattern in halide perovskites.

Table S5. Key parameters of critical points derived from the topological analysis of $Rb_3Bi_2Br_9$, including bond length, electron density (ρ), Laplacian of electron density ($\nabla^2\rho$), potential energy density (v), Lagrangian kinetic energy density (G), and total energy density (H) at the bond critical points.

Bond	Length (Å)	ρ ($\times10^{-3}$)	$\nabla^2\rho$ ($\times10^{-2}$)	G ($\times10^{-3}$)	v ($\times10^{-3}$)	H ($\times10^{-3}$)	$\|v\|/G$
Rb–Br	3.469	9.94	3.39	7.14	-5.81	1.33	0.81
Rb–Br	3.600	7.72	2.63	5.44	-4.30	1.14	0.79
Rb–Br	3.620	7.36	2.53	5.19	-4.06	1.13	0.78
Bi–Br	2.757	52.7	5.14	31.3	-40.0	-8.67	1.28
Bi–Br	2.748	53.0	9.09	31.5	-40.3	-8.79	1.28
Bi–Br	2.752	52.4	9.03	31.1	-39.5	-8.48	1.27
Bi–Br	2.765	51.5	8.80	30.2	-38.5	-8.24	1.27
